# The hereditary mutation G51D unlocks a distinct fibril strain transmissible to wild-type α-synuclein

**DOI:** 10.1038/s41467-021-26433-2

**Published:** 2021-10-29

**Authors:** Yunpeng Sun, Houfang Long, Wencheng Xia, Kun Wang, Xia Zhang, Bo Sun, Qin Cao, Yaoyang Zhang, Bin Dai, Dan Li, Cong Liu

**Affiliations:** 1 Interdisciplinary Research Center on Biology and Chemistry, Shanghai Institute of Organic Chemistry, Chinese Academy of Sciences, 201210 Shanghai, China; 2 University of Chinese Academy of Sciences, 100049 Beijing, China; 3 School of Life Science and Technology, ShanghaiTech University, 201210 Shanghai, China; 4 Bio-X Institutes, Key Laboratory for the Genetics of Developmental and Neuropsychiatric Disorders, Ministry of Education, Shanghai Jiao Tong University, 200030 Shanghai, China; 5 Institute of Nano Biomedicine and Engineering, Department of Instrument Science and Engineering, School of Electronic Information and Electrical Engineering, Shanghai Jiao Tong University, 200210 Shanghai, China

**Keywords:** Structural biology, Intrinsically disordered proteins, Cryoelectron microscopy

## Abstract

α-Synuclein (α-Syn) can form different fibril strains with distinct polymorphs and neuropathologies, which is associated with the clinicopathological variability in synucleinopathies. How different α-syn fibril strains are produced and selected under disease conditions remains poorly understood. In this study, we show that the hereditary mutation G51D induces α-syn to form a distinct fibril strain in vitro. The cryogenic electron microscopy (cryo-EM) structure of the G51D fibril strain was determined at 2.96 Å resolution. The G51D fibril displays a relatively small and extended serpentine fold distinct from other α-syn fibril structures. Moreover, we show by cryo-EM that wild-type (WT) α-syn can assembly into the G51D fibril strain via cross-seeding with G51D fibrils. Our study reveals a distinct structure of G51D fibril strain triggered by G51D mutation but feasibly adopted by both WT and G51D α-syn, which suggests the cross-seeding and strain selection of WT and mutant α-syn in familial Parkinson’s disease (fPD).

## Abstract (web summary)

G51D mutation of α-synuclein (α-syn) causes a subset of familial Parkinson’s disease that is characterized by an early onset and rapid progression of the disease. Here, the authors present the cryo-EM structure of full-length G51D α-syn fibrils that is distinct from other known α-syn fibril structures, and they show that G51D fibrils can cross-seed wild-type (WT) α-syn and that these cross-seeded WT fibrils replicate the G51D fibril structure.

## Introduction

α-Syn is a highly abundant presynaptic protein in neurons. It forms amyloid aggregation and condenses into Lewy bodies (LB) and Lewy neurites (LN) which are histological hallmarks of Parkinson’s disease (PD) and other synucleinopathies^1–3^. Mounting evidence has shown that amyloid fibrils of α-syn serve as a key pathological entity that may impair protein homeostasis, sequester mitochondria, and mediate cell-to-cell transmission and spread of the α-syn pathology during the disease progression^4–7^. Strikingly, α-syn can form various fibril strains both in vitro and in vivo^8–14^. Different α-syn fibril strains exhibit distinct neuropathologies. Therefore, different strains may be involved in different types of synucleinopathies with a wide spectrum of heterogeneous clinical symptoms^14–18^, including PD, multiple system atrophy (MSA), dementia with Lewy bodies (DLB)^17,19,20^. Indeed, the α-syn fibrils derived from the brains or the cerebrospinal fluid (CSF) of PD and MSA patients showed not only different fibril polymorphs (strains) but also distinct pathological properties such as cell-to-cell propagation and neurotoxicity^15,17,21^. Thus, it is important to understand how α-syn selects to adopt one fibril strain but not the other under certain circumstances.

Recent cryo-EM studies have shown that hereditary mutations (e.g., H50Q, E46K, and A53T) and disease-associated phosphorylation (pY39) of α-syn can induce distinct fibril strains^22–26^. This implies that mutation and post-translational modification (PTM) may play important roles in α-syn strain selection. In this study, we focused on the hereditary mutation G51D which is one of the eight disease-causing mutations identified in fPD and explored its role in α-syn fibril strain formation and selection. G51D mutation was firstly described from a French family with a Parkinsonian-pyramidal syndrome^27^. An unusual PD phenotype of G51D was characterized by early disease onset, rapid progression, and death within a few years^27,28^. Previous studies showed that G51D α-syn fibrils are more toxic than WT α-syn fibrils in cell models^29,30^. Intriguingly, gene *SNCA* of the fPD patients with G51D mutation are heterozygous^27^, so both WT and G51D α-syn co-exist in the patients. Therefore, it is important to investigate whether and how the G51D and WT α-syn may influence the fibril strain selection of each other.

In this work, we determined the cryo-EM structure of G51D fibril strain prepared in vitro at a resolution of 2.96 Å. The G51D strain displays potent neuropathology and exhibits a distinct structure from all known α-syn strains. Moreover, we found that the G51D strain can induce WT α-syn monomer to form amyloid fibril with a similar structure to the G51D strain via cross-seeding. Our work demonstrates that the G51D α-syn fibril structure presented here can be adopted by both G51D and WT α-syn, which indicates that the mutant and WT α-syn may concertedly accelerate the disease progression in fPD with G51D mutation.

## Results

### G51D α-syn forms neuropathological fibrils with a right-handed helical twist

**Fig. 1:**
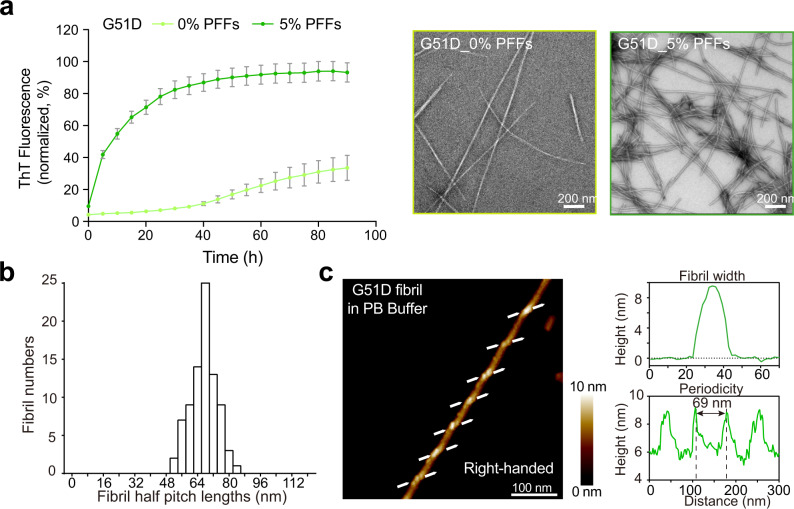
Characterization of the fibrils formed by G51D α-syn. **a** ThT kinetic assay of the G51D fibril formation with (colored in dark green) and without PFFs (5 mol%) (colored in light green) in PB Buffer (50 mM phosphate buffer, pH 7.0, 50 mM NaCl). Data are shown as mean ± s.d., *n* = 5 independent samples (left panel). The fibrils formed at the endpoint of the ThT kinetic assay were characterized by NS-TEM (right panel). Scale bar: 200 nm. **b** Histogram of half-pitch lengths of G51D fibrils (0% PFFs) measured from NS-TEM images (*n* = 83). **c** AFM image of a G51D fibril. The arrows at both sides of the fibril indicate the starting points of the fibril protrusions to clarify the handedness. Images were acquired with a 1.45 Hz rate at 512 × 512 pixels. Fibrils formed by G51D in >3 independent experiments provide reproducible images. The fibril width and periodicity were analyzed by the Nanoscope software.

To investigate how G51D mutation influences the fibril formation of α-syn, we overexpressed and purified full-length G51D α-syn with N-terminal acetylated. By performing the Thioflavin T (ThT) kinetic assay and negative-staining transmission electron microscopy (NS-TEM), we showed that G51D α-syn spontaneously forms long and unbranched amyloid fibrils with a lag time of ~16 h in 50 mM phosphate buffer, pH 7.0, 50 mM NaCl (referred to as PB buffer) (Fig. 1a). The G51D fibrils are of good homogeneity with half pitches in the range of 67.0 ± 9.6 nm (Fig. 1b). We confirmed that the G51D fibrils are formed by full-length G51D α-syn by SDS–PAGE, Western blot, and matrix-assisted laser desorption/ionization time-of-flight mass spectrometry (MALDI-TOF MS) (Supplementary Fig. 1). In the presence of preformed fibrils (PFFs) of G51D α-syn, the G51D α-syn monomer rapidly forms fibril with greatly shortened lag time and reaches a plateau in 55 h (Fig. 1a). The atomic force microscopy (AFM) further reveals that the G51D fibril features a right-handed helical twist (Fig. 1c and Supplementary Fig. 2), which is distinct from all known α-syn fibrils (left-handed) except for the E46K fibril^9–14,22–26^. To further confirm the causative role of G51D mutation in the structural change, we prepared fibrils with WT α-syn under the same condition (i.e., in the PB buffer). AFM and NS-TEM images showed that WT α-syn formed a mixture of three polymorphs including straight polymorph (~93%), and two left-handed twist polymorphs (~3.6% and ~2.6%, respectively) in PB buffer, which are all distinct from the right-handed twist morphology of the G51D fibril formed under the same condition (Supplementary Fig. 2).

We next sought to explore the physiochemical and pathological properties of the G51D fibrils and compared it with a previously well-characterized fibril polymorph formed by WT α-syn (referred to as WT_1a_)^11^. We firstly measured the fibril stability by using a proteinase K (PK) digestion assay. The result showed that the G51D PFFs are digested markedly faster than the WT_1a_ PFFs (Supplementary Fig. 3a). We further investigated the fibril stability by sonication. The NS-TEM images showed that the G51D fibrils were fragmented into shorter pieces than the WT_1a_ fibrils upon sonication (Supplementary Fig. 3b). Moreover, the cellular viability results showed that the G51D PFFs exhibit stronger cellular toxicity to SH-SY5Y cells than the WT_1a_ PFFs (Supplementary Fig. 4a). Finally, we examined the neuronal propagation capability of G51D by using a well-established primary neuron assay^31^. Notably, the G51D PFFs induced significantly more pathological aggregation of α-syn in neurons than the WT_1a_ PFFs (Supplementary Fig. 4b, c). Taken together, these data indicate that the G51D fibrils prepared in this work display a distinct fibril polymorph with decreased stability and potent neuropathology.

### Cryo-EM structure determination of the G51D α-syn fibril

**Fig. 2:**
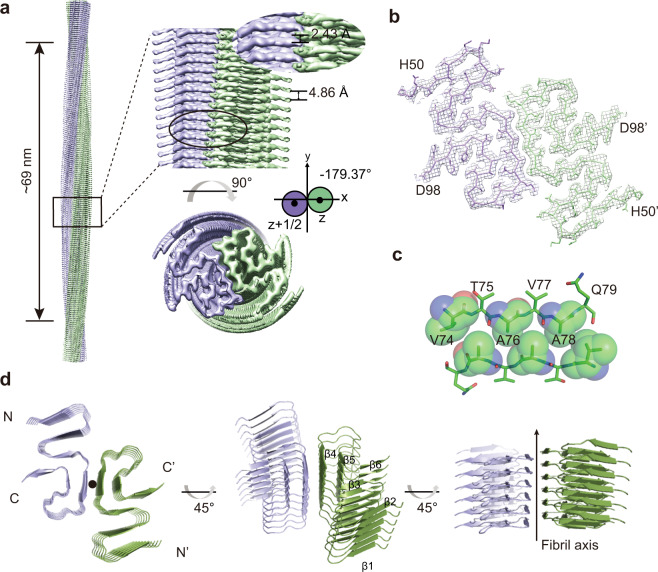
Cryo-EM structure of the G51D α-syn fibril. **a** Cryo-EM density map of G51D α-syn fibril. Fibril parameters including half-pitch, twist angle, and rise are marked. The two protofilaments are colored in medium purple and forest green, respectively. The map was prepared by UCSF Chimera v1.13. **b** Cross-section view of the density map with a built-in structure model. **c** The protofilament interface of G51D fibril is shown in sticks. Residues involved in the inter-protofilament interactions are indicated in spheres. **d** Views of six layers of G51D α-syn fibrils are shown in the cartoon. The fibril axis is indicated. The β-strands are numbered and labeled accordingly.

We next sought to determine the atomic structure of the G51D fibril by using cryo-EM. We collected 1869 micrographs from a glow-discharged holey carbon grid containing well-dispersed G51D fibrils. One major fibril polymorph was identified with a population of over 95% of the total 88,917 fibril segments with a 1024-pixel box size from the two-dimensional (2D) classification. Based on the high-quality cryo-EM density map acquired, we managed to build a structural model of G51D fibril with an overall resolution of 2.96 Å (Supplementary Fig. 5). The G51D fibril is composed of two intertwining protofilaments with a right-handed helix (Fig. 2a). The subunits within each protofilament stack along the fibril axis with a helical twist of 1.26° and helical rise of 4.86 Å (Fig. 2a and Table 1). Residues 50–98 of α-syn form the fibril core (FC), and the rest of the residues in the N- and C-terminal regions are relatively flexible in the fibril and invisible by cryo-EM imaging (Fig. 2b). Residues 74–79 from opposing α-syn subunits form a highly complementary and dry interface for zippering the two protofilaments together (Fig. 2c).

**Table 1:** Statistics of cryo-EM data collection and refinement.

Name	G51D fibril	WT_51cs_ fibril	WT_wts_ fibril
PDB	7E0F	n/a	n/a
EMDB	EMD-30931	n/a	n/a
*Data collection*			
Magnification	22,500	22,500	22,500
Pixel size (Å)	1.06	1.06	1.06
Defocus range (μm)	−1 to −2	−1 to −2	−1 to −2
Voltage (kV)	300	300	300
Detector	K3	K3	K3
Microscope	Titan Krios	Titan Krios	Titan Krios
Exposure time (s per frame)	0.097	0.097	0.097
Number of frames	32	32	32
Total dose (e− per Å^2^)	55	55	55
*Reconstruction*			
Micrographs	1869	725	1056
Manually picked fibrils	28,988	12,428	7903
Box size (pixel)	288	686	686
Inter-box distance (Å)	30.5	72.7	72.7
Initial particle images (no.)	349,716	90,963	74,598
Final particle images (no.)	213,348	40,587	16,120
Map resolution (Å)	2.96	n/a	n/a
FSC threshold	0.143	n/a	n/a
Map resolution (Å)	3.15	n/a	n/a
FSC threshold	0.5	n/a	n/a
Map sharpening B-factor (Å^2^)	−144.573	n/a	n/a
Helical rise (Å)	2.43	n/a	n/a
Helical twist (°)	−179.37	n/a	n/a
*Refinement*			
Initial model used	6L4S	n/a	n/a
non-hydrogen atoms	2, 022	n/a	n/a
Protein residues	294	n/a	n/a
Ligands	0	n/a	n/a
r.m.s.d. bond lengths	0.016	n/a	n/a
r.m.s.d. bond angles	0.986	n/a	n/a
All-atom clash score	9.76	n/a	n/a
Rotamer outliers	0.00%	n/a	n/a
Ramachandran outliers	0.00%	n/a	n/a
Ramachandran allowed	12.77%	n/a	n/a
Ramachandran favored	87.23%	n/a	n/a

n/a, not applicable.

### Structure comparison of WT, G51D, and E46K α-syn fibrils

**Fig. 3:**
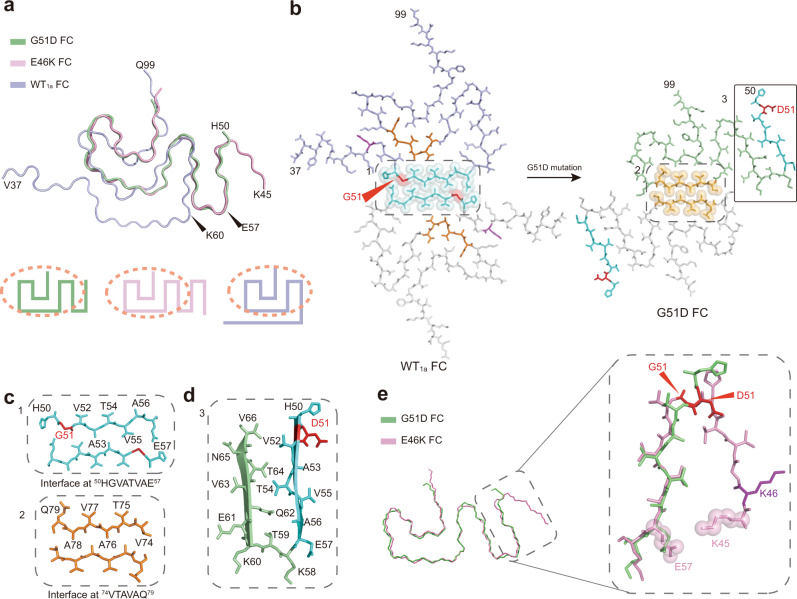
Structure comparison of G51D, WT_1a,_ and E46K fibrils. **a** Overlay of the structures of every single α-syn subunit from the G51D, E46K, and WT_1a_ fibrils. G51D fibril is in green; WT_1a_ fibril is in slate; E46K fibril is in pink. The region with a similar structure shared by three different FC is circled on topology diagrams. **b** Rearrangement of α-syn fibril structure triggered by G51D mutation. Residues involved in the protofilament interfaces are presented in spheres. The protofilament interface of WT_1a_ fibril is colored in cyan; the interface of the G51D fibril is colored in orange. D51 and G51 are highlighted in red. E46 and K46 are in purple. **c** Zoom-in views of the WT_1a_ and G51D fibril interfaces. **d** The β-hairpin (residues 50–66) formed in the G51D fibril structure is zoomed in with D51 in red. **e** Overlay of the structures of every single α-syn subunit from the G51D and E46K fibrils. G51D mutation disrupts the β-turn formed of residues 45–57 formed in the E46K fibril. G51 and D51 are highlighted in red. K46 is highlighted in purple.

The G51D FC consists of six β-strands (β_1_–β_6_) which arranges into an extended serpentine fold (Fig. 2d). The overall structure of G51D fibril is distinct from all the 19 previously determined α-syn fibril structures formed by either WT α-syn or variants^9–14,22–26^, although the G51D fibril exhibits structural similarity with WT polymorph 1a (WT_1a_, PDB ID: 6A6B) and E46K fibril (PDB ID: 6L4S)^9,25^ (Fig. 3a). In particular, residues 60–98 form a similar architecture in G51D, WT_1a_, and E46K fibrils (Fig. 3a). However, both G51D and E46K fibrils feature a smaller and more loose FC than that of the WT_1a_ (Fig. 3a, b). In the WT_1a_ structure, G51 is involved in the steric-zipper interface of the two protofilaments formed by residues 50–57 (^50^HGVATVAE^57^), which is assumed to be disrupted by G51D mutation (Fig. 3b, c). Instead, this segment with G51D mutation flips 180° perpendicular to the fibril axis and forms a β-hairpin structure with residues 60–66 in the mutant fibril (Fig. 3d). Meanwhile, V74, A76, and A78 that used to be buried inside the WT_1a_ FC are exposed in G51D FC and form a homotypical steric-zipper interface to bundle the two protofilaments together (Fig. 3b, c).

In the E46K fibril structure, residues 45–53 form an additional β-turn, which attaches to the β-hairpin by a salt bridge formed by E57 and K45. G51 is essential in forming the β-turn (Fig. 3e). Thus, the replacement of Gly to Asp disrupts the β-turn formation and leaves the entire N-terminal (residues 1–49) flexible and invisible from cryo-EM (Fig. 3b, e). Therefore, despite that G51D FC shares structural similarity with WT_1a_ and E46K FCs, G51D mutation excludes the formation of either WT_1a_ or E46K fibril structures.

### WT α-syn forms G51D fibril strain by cross-seeding

**Fig. 4:**
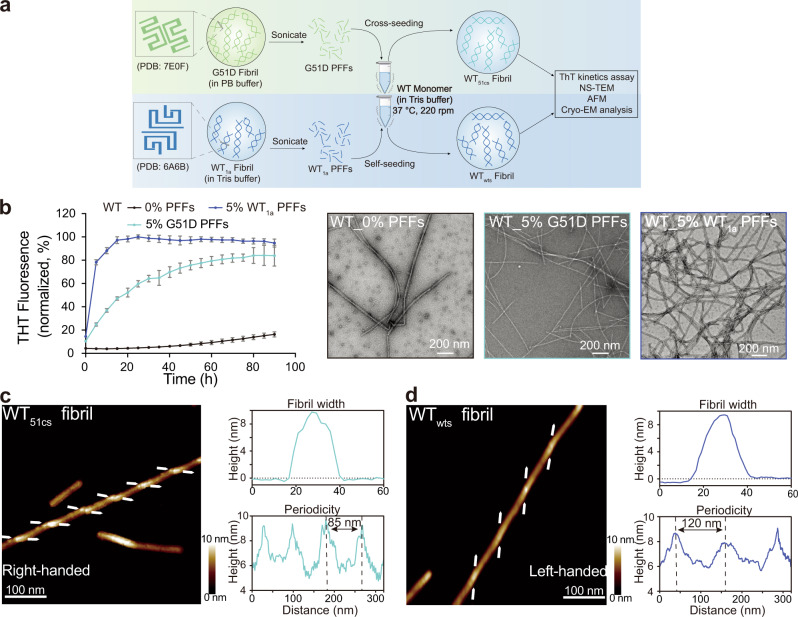
Seeding of WT α-syn by both G51D and WT α-syn PFFs. **a** Schematic diagram of the seeding process. PDB IDs of WT_1a_ and G51D fibrils used for the seeding experiments are provided in parentheses. Buffer (50 mM Tris, pH 7.5, 150 mM KCl, 0.05% NaN_3_) is referred to as Tris buffer. Buffer (50 mM phosphate buffer, pH 7.0, 50 mM NaCl, 0.05% NaN_3_ buffer) is referred to as PB buffer. The schematic diagram was created from scratch. **b** Fibrillation of WT α-syn alone (colored in black) or in the presence of 5 mol% WT PFFs (colored in purple) and G51D PFFs (colored in cyan), respectively. Data are shown as mean ± s.d., *n* = 5 independent samples. The fibrils sampled at the end of the ThT kinetics assay were imaged by NS-TEM (right panel). Scale bar: 200 nm. **c** and **d** AFM images of the WT_51cs_ (**c**) and WT_wts_ fibrils (**d**). The images represent reproducible results in three independent experiments. The arrows at both sides of the fibril indicate the starting points of the fibril protrusions to clarify the handedness. Images were acquired with a 1.45 Hz rate at 512 × 512 pixels. The fibril width and periodicity were analyzed by the Nanoscope software.

Since both WT and G51D α-syn co-exist in the fPD patients carrying heterozygous G51D mutation^27^, we next asked whether WT α-syn can form the G51D fibril strain in the presence of the preformed G51D fibril PFFs (Fig. 4a). As previously reported, WT α-syn forms left-handed fibrils featuring WT_1a_ structure under the condition of buffer (50 mM Tris, pH 7.5, 150 mM KCl, 0.05% NaN_3_)^11^. Under the same fibrillation condition, we examined the seeding effect of WT_1a_ PFFs and G51D PFFs for WT α-syn fibrillation, respectively. The result showed that both WT and G51D PFFs exhibit potent seeding activity in inducing fibrillation of WT α-syn monomer monitored by NS-TEM and the ThT kinetics assay (Fig. 4b). Strikingly, AFM measurement revealed that the WT α-syn fibril cross-seeded by G51D PFFs (termed as WT_51cs_ fibril) features a right-handed twist same as its template G51D fibril (Fig. 4c). While the fibril seeded by WT_1a_ PFFs (termed as WT_wts_ fibril) displays a left-handed twist and inherited the fibril morphology of its template-WT_1a_ fibril (Fig. 4d and Supplementary Fig. 6).

**Fig. 5:**
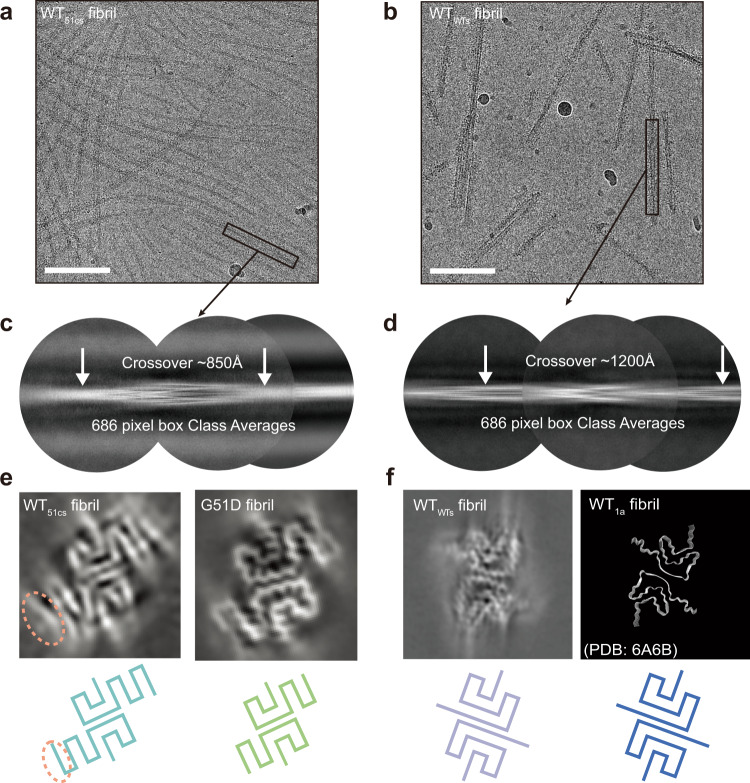
Cryo-EM study of WT_51cs_ and WT_wts_ strains. Cryo-EM micrographs of WT_51cs_ strain (**a**) and WT_wts_ strain (**b**) are shown. The images represent reproducible results in three independent experiments. Scale bar = 100 nm. 686-pixel box size 2D class averages comprising a helical crossover of WT_51cs_ strain (**c**) and WT_wts_ strain (**d**) are shown and used to determine crossover distance. **e** Central slice of the 3D map of the WT_51cs_ and G51D strains. The additional electron density of the WT_51cs_ strain compared to the G51D strain is circled. **f** Central slice of the 3D map of the WT_wts_ (left). Structural models of WT_1a_ strain (PDB ID: 6A6B) are shown on the right. The topology diagram of α-syn for each strain is shown (bottom).

We further explored the structures of the WT_51cs_ and WT_wts_ fibrils by using cryo-EM (Fig. 5a–d). We fixed the WT_51cs_ and WT_wts_ fibril samples on the carbon grids in liquid ethane and collected 725 and 1063 cryo-EM micrographs, respectively. 2D classifications showed one dominant species of the WT_51cs_ fibril sample with a population of ~97% of the total 44,870 fibril segments with 1200-pixel box size. WT_wts_ fibril sample contains the main species with a population of ~91% of the total 44,909 fibril segments with a 1024-pixel box size. Notably, three-dimensional (3D) reconstructions revealed that WT_51cs_ exhibits a highly similar FC structure as that of the G51D fibril, indicating that the structure of G51D fibril is inherited by WT α-syn upon cross-seeding (Fig. 5c, e). Of note, compared to G51D, WT_51cs_ showed additional electron density adjacent to the β-hairpin (residues 51–66) (Fig. 5e). The density may represent the β-turn consisting of residues 45–53 as observed in the E46K strain. As for the WT_wts_ fibril, 3D reconstructions confirmed that WT_wts_ recapitulates the architecture of its template WT_1a_ fibril (Fig. 5d, f). Taken together, our results demonstrate that G51D mutation is sufficient but not necessary for forming the structure of the G51D fibril strain. WT α-syn can also adopt the G51D-like fibril structure in the presence of G51D fibril seeds.

## Discussion

Structural polymorphs have been widely observed in amyloid fibrils formed by amyloid proteins such as α-syn, Tau, and Aβ^18,32–36^. Different fibril polymorphs exhibit distinct physiochemical and pathological properties, which are believed to underlie the clinicopathological variability of the associated diseases^17,19,37–39^. In this study, we found that the PD familial mutation G51D can trigger α-syn to form a new fold assembling into an unusual right-handed fibril in PB buffer (50 mM phosphate buffer, pH 7.0, 50 mM NaCl). The G51D fibril structure provides a structural explanation for how a single mutation G51D excludes the formation of other fibril strains (e.g. WT_1a_ and E46K strains) and induces the formation of a new strain. It is worthwhile to note that G51D α-syn can also form polymorphic fibrils under different conditions. We previously observed that G51D α-syn can form a straight fibril in the Tris buffer containing 50 mM Tris, pH 7.5, 150 mM KCl, which is morphologically different from the left-handed twist fibril formed by WT α-syn under the same condition (Supplementary Fig. 2)^11^. Unfortunately, due to the technical limitation of cryo-EM, we cannot yet solve untwisted fibril structures so far. Nevertheless, it will be interesting to investigate how G51D induces different fibril strains as well as their distinct pathological roles in disease.

In addition to determining the G51D fibril structure, we further demonstrate that WT α-syn can also form the structure of the G51D fibril strain by cross-seeding. fPD patients with G51D mutation carry heterozygous *SNCA* missense mutation, which means that both WT and G51D α-syn exist in the patients’ brains. Since the G51D fibril can efficiently cross-seed WT α-syn and induce it to form a G51D-like fibril structure, it is possible that the G51D strain may spread to the WT α-syn and jointly accelerate the disease progression in fPD. In addition to the scenario of cross-seeding, it might also be biologically relevant to investigate whether WT and G51D α-syn can co-aggregate to form amyloid fibrils. We thus mixed WT and G51D α-syn monomers at a 1:1 molar ratio (termed as WT + G51D), and monitored the aggregation kinetics and fibril formation by ThT assay and NS-TEM. The result showed that mixing of WT and G51D α-syn generally enhanced the amyloid aggregation compared to individual proteins (Supplementary Fig. 7). Furthermore, mass spectrometry results confirmed that the WT + G51D fibrils indeed consist of both WT and G51D α-syn (Supplementary Fig. 8). However, it is unclear whether WT and G51D proteins formed separate fibrils or hybrid fibrils. Future structure determination on WT + G51D fibrils may provide an answer. Moreover, other PD familial mutations such as E46K and A53T have been reported to induce the formation of distinct α-syn fibril strains^23–25^. Further study is required to systemically investigate the cross-seeding and co-aggregation activities and their mutual influences in fibril strain selection and α-syn pathology under disease conditions.

## Methods

### Purification of recombinant WT and G51D α-syn

Preparation of full-length WT and G51D α-syn follows the similar protocol described previously^11^. Briefly, the genes encoding WT and G51D α-syn were inserted into the pET22 vector. G51D point mutation gene was generated based WT α-syn gene by PCR kit (TransGen Biotech, Cat. # AP101-11). Primer sequences used in this work are listed in Supplementary Table 1. α-Syn was co-expressed with yeast N-acetyltransferase complex B in BL21 (DE3) cells (TransGen Biotech, Cat. # CD601-02) to obtain the N-terminally acetylated α-syn protein^40^. Cells were harvested after protein expression at 37 °C for 4 h with 1 mM isopropyl-1-thio-d-galactopyranoside (IPTG). Then, cells were lysed by sonication in 50 mM Tris–HCl, pH 8.0, 1 mM phenylmethylsulfonyl fluoride, 1 mM EDTA. The supernatant was obtained by centrifugation at 15,000 × *g* for 25 min. Then the supernatant was processed by boiling at 100 °C for 10 min, streptomycin treatment (20 mg/ml), pH adjustment to 3.5 using 2 M HCl, and dialysis 50 mM Tris–HCl (pH 8.0) overnight in turn. Anion exchange column (GE Healthcare, 17-5156-01) and Superdex 75 (GE Healthcare, 28-9893-33) were then used to purify α-syn protein in high purity. For the anion exchange column, buffer (50 mM Tris–HCl, 1 M NaCl, pH 8.0) was used to elute protein with a gradient (0–60%). Finally, an online EASY-nL-LC 1000 coupled with an Orbitrap Q-Exactive HF mass spectrometer was used to validate that the α-syn protein is indeed acetylated.

### Preparation of WT_1a_ and G51D α-syn fibril strains

Recombinant WT (100 μM, in 50 mM Tris, pH 7.5, 150 mM KCl, 0.05% NaN_3_ buffer) and G51D (100 μM, in 50 mM phosphate buffer, pH 7.0, 50 mM NaCl, 0.05% NaN_3_ buffer) were shaking at 37 °C, 900 rpm in ThermoMixer (Eppendorf) for 7 days, respectively. α-Syn preformed fibril seeds (PFFs) were obtained by sonication with 20% power for 15 times (1 s per time, 1 s per interval) on ice by JY92-IIN sonicator. Then, 100 μM WT and G51D α-syn monomer were incubated in the presence of α-syn PFFs (0.5 mol%, concentration by monomer equivalent) at 900 rpm, 37 °C for a week. The residual soluble α-syn in the supernatant was removed after pelleting the fibrils. The pellets were suspended with buffer to 100 μM (equivalent to monomer concentration). The fibril samples were further used for NS-TEM, AFM, cryo-EM sample preparation, PK assay, sonication, and primary neuron treatment.

### ThT kinetic assay

Seeding of WT and G51D monomer by α-syn PFFs was conducted by using ThT assay. 50 μM α-syn WT (in 50 mM Tris, pH 7.5, 150 mM KCl, 0.05% NaN_3_) monomer was incubated with the WT_1a_ α-syn PFFs and G51D α-syn PFFs (5 mol%, equivalent to monomer concentration) with 10 μM ThT in the reaction mixture, separately. Similarly, 50 μM α-syn G51D (in 50 mM phosphate buffer, pH 7.0, 50 mM NaCl, 0.05% NaN_3_) monomer was incubated with the G51D (5 mol%, equivalent to monomer concentration) with 10 μM ThT in the reaction mixture. A Fluoroskan Ascent microplate reader (Thermo Scientific) was used to test reactions performed in a 384-well optical plate (Thermo Scientific) in triplicate, with 440 nm excitation wave-length and 485 nm emission wave-length, a bottom read. Graphing was performed with GraphPad Prism 6. The data shown in each ThT experiment are mean ± s.d., *n* = 5 independent samples.

A co-aggregation experiment of α-syn WT and G51D was conducted by using ThT assay. 100 μM α-syn WT, 100 μM G51D monomer or mixed monomer (50 μM WT + 50 μM G51D) was incubated with 10 μM ThT in the reaction mixture in PB buffer (50 mM phosphate buffer, pH 7.0, 50 mM NaCl, 0.05% NaN_3_) or Tris Buffer (50 mM Tris, pH 7.5, 150 mM KCl, 0.05% NaN_3_). A Fluoroskan Ascent microplate reader (Thermo Scientific) was used to test reactions performed in a 384-well optical plate (Thermo Scientific) in triplicates, with 440 nm excitation wavelength and 485 nm emission wavelength, a bottom read. Graphing was performed with GraphPad Prism 6. The data shown in each ThT experiment are mean ± s.d., *n* = 3 independent samples.

The sample from the ThT assay was further characterized by NS-TEM. 50 μl aqueous solution of each sample from the ThT assay was pelleted by centrifugation (14,462 × *g*, 25 °C, 45 min). SDS-loading buffer was added into 45 μl supernatant and boiled for 10 min. The pellet was washed by phosphate-buffered saline (PBS) and dissolved in 45 μl buffer, then sonicated for 5 min and boiled for 30 min. Lastly, the solution was boiled in the SDS-loading buffer for 10 min. The supernatant samples and dissolved pellet samples were loaded on 4–20% Bis–Tris gels (GenScript), respectively. The gels were stained by Coomassie brilliant blue and images were acquired and analyzed with Image Lab 3.0 (Bio-Rad).

### Atomic force microscopy

Fibril samples were mounted on mica for 3 min, rinsed gently with water, and dried with nitrogen flow. Images were captured by Nanoscope V Multimode 8 (Bruker) with SNL-10 probes (a constant of 0.35 N m^−1^) on ScanAsyst air mode. Images were acquired with a 1.5 Hz rate at 512 × 512 pixels and analyzed on the Nanoscope software.

### Negative-staining transmission electron microscopy

Samples were prepared by loading 5 μl of fibril solution onto a glow-discharged 200 mesh carbon support film (Zhongjingkeyi Technology Co., Ltd., Beijing). The samples were held for 45 s and washed with double-distilled water followed by 3% uranyl acetate. The grid was then stained with 3% uranyl acetate for 45 s and allowed to dry in air. The samples were imaged by a Tecnai T12 microscope (FEI).

### PK digestion of α-syn PFFs

WT_1a_ or G51D α-syn PFFs were prepared by sonication at 20% power for 15 times (1 s per time, 1 s per interval) on ice by JY92-IIN sonicator. α-Syn PFFs (3 mg ml^−1^, 25 μl, in PBS, pH 7.4) were incubated with proteinase K (final concentration 0.5 and 1.5 μg ml^−1^, Invitrogen) at 37 °C for 10, 30, 60 and 200 min. 1 mM PMSF was added to samples to stop the reaction. Then samples were boiled with an SDS-loading buffer for 15 min and loaded on 4–20% Bis–Tris gels (GenScript). The gels were stained by Coomassie brilliant blue and images were recorded and analyzed with Image Lab 3.0 (Bio-Rad). Graphing was performed with GraphPad Prism 6. The data shown in are mean ± s.d., *n* = 3 independent samples.

### Characterization of fibril fragmentation

20 μM WT_1a_ or G51D α-syn fibrils were sonicated with 20% power 2 times and 15 times on ice by JY92-IIN sonicator. And 5 μM WT_1a_ or G51D α-syn PFFs (under the corresponding condition) were characterized by NS-TEM.

### Cell viability assay

SH-SY5Y cells were cultured to test the cytotoxicity of G51D and WT1a PFFs with a CCK-8 kit. SH-SY5Y cells cultured in a 96-well plate were treated with α-syn PFFs at final concentrations of 0.01, 0.1, and 1 μM for 24 h. Then cell viability was tested with the CCK-8 kit following the manufacturer’s protocol. Briefly, CCK-8 solution (10 μl/well) was added to each well. The absorbance of the plate was measured at 450 nm after incubating for 30 min. The data were analyzed with GraphPad Prism 6.

### Primary neuronal culture experiments

Primary cortical neurons were dissected from the cortex of embryonic day (E) 16–E18 Sprague Dawley rats (Shanghai SIPPR BK Laboratory Animals Ltd, China) embryos as previously described^41^. In brief, primary neurons were seeded onto coverslips previously coated with poly-D-lysine (PDL) in 24-well plates (150,000 cells/coverslip). At 8-day in vitro (DIV), neurons were treated with PBS and 100 nM (final concentration) WT_1a_ or G51D α-syn PFFs, and collected for immunofluorescence at 10/14-day post-treatment. The primary antibodies used in the assay were the phospho-α-synuclein (S129) antibody (Abcam, cat. no. ab51253) and the MAP2 antibody (Abcam, cat. no. ab5392). The antibodies were diluted at a ratio of 1:1000. The intensity of confocal images was analyzed by Image J 2.0.0. The secondary antibodies included goat anti-rabbit IgG Alexa Fluor 568 (Abcam, cat. no. ab175471) and goat anti-chicken Alexa Fluor 488 (Thermo Fisher, cat. no. A-11039). All rat experiments were performed followed the protocols approved by the Animal Care Committee of the Interdisciplinary Research Center on Biology and Chemistry (IRCBC), Chinese Academy of Sciences (CAS). There are three samples in each group.

### Purity characterization of G51D fibril

To characterize the purity of α-syn fibrils, we dissolved the fibrils with buffer (50 mM Tris, pH 8.0, 150 mM NaCl, 1% Triton X-100, 2% SDS) and performed SDS–PAGE. The gels were stained by Coomassie brilliant blue and images were acquired and analyzed with Image Lab 3.0 (Bio-Rad). In addition, the fibrils were also detected by western blot. The monomer and fibrils were immunoblotted by anti-α-synuclein antibodies: 2642S (Cell signaling) and ab138501 (Abcam), respectively. The antibodies were diluted at a ratio of 1:1000.

### MALDI-TOF MS

A droplet of 1 μl solution containing G51D monomer or dissolved fibrils was mixed with 1 μl of the sinapinic acid matrix (10 mg of sinapinic acid per ml, a 70:30 water/acetonitrile (ACN) with 0.1% trifluoroacetic acid). Deposit 1 μl of final mix onto a MALDI stainless steel target and allow to air dry at room temperature. A 5800 MALDI-TOF/TOF mass spectrometer (AB SCIEX, Framingham, MA, USA) in linear positive mode with a mass range from *m*/*z* 2500 to 20,000 was used for MALDI-TOF MS measurements and analyses.

### LC–MS/MS analysis

Excise bands of dissolved fibril samples loaded on 4–20% Bis–Tris gels with a clean scalpel and cuts gel bands into cubes. Gel pieces were then destained with ammonium bicarbonate/ACN (1:1, vol/vol), shrink with neat ACN, and then saturated gel pieces with trypsin. After digestion with trypsin by incubating samples overnight at 37 °C, extract peptide digestion products by incubating samples with extraction buffer (1:2 (vol/vol) 5% formic acid (FA)/ACN for 15 min at 37 °C in a shaker. After centrifugation at 16,000 × *g* and 4 °C for 15 min, the clear supernatants were collected, dry down in a vacuum centrifuge, and then resuspended in 0.1% (vol/vol) FA for further LC–MS/MS analysis.

The peptide mixture was analyzed using an Orbitrap Fusion mass spectrometer coupled to an online EASY-nL-LC 1000 system. Mobile phase A consisted of 0.1% FA, 2% ACN, and 98% H_2_O, and mobile phase B consisted of 0.1% FA, 2% H_2_O, and 98% ACN. A 60 min gradient (mobile phase B: 3% at 0 min, 8% at 5 min, 20% at 46 min, 30% at 54 min, 95% at 55 min, and 95% at 60 min) was used at a static flow rate of 300 nL/min. The data were acquired in a data-dependent (top 20) mode. High-energy collisional dissociation (HCD) was used to fragment the precursor peptides, and the resulting fragment ions were measured in the ion trap analyzer.

### Cryo-EM data collection

A solution containing G51D or WT_51cs_ fibril samples was applied to glow-discharged holey carbon Cu Quantifoil grids (R2/1, 300 mesh) and then plunge-frozen in liquid ethane after blotting with filter paper using Vitrobot Mark IV(FEI). Cryo-EM micrographs with a defocus from −1 to −2 μm were collected on a Gatan K3 direct detector in super-resolution mode on a Titan Krios transmission electron microscope (FEI) operated at 300 kV. 32 movies were recorded per micrograph was a record with a pixel size of 1.06 Å pixel^−1^ using a dose of 55 e^−^ Å^−2^. Automated cryo-EM data collection was performed by Serial EM software^42^.

### Imaging processing, reconstruction, and model building

MotionCorr2^43^ was used to correct beam-induced motion of movie frames with dose weighting while CTFFIND4.1.8^44^ was used to estimate the contrast transfer function. All filaments were picked manually using the manual picking method of RELION3.0^45^. All subsequent steps of helical reconstruction were carried out using RELION 3.0.

### G51D dataset

28,989 filaments were manually picked from 1869 micrographs. G51D segments were first extracted using 1024-pixel box size with an inter-box distance of 109 Å and used for subsequent reference-free 2D classification with a decreasing in-plane angular sampling rate from 12° to 1° and a *T* = 2 regularization parameter to estimate the fibril pitch and helical parameters. Filaments containing selected segments with 1024-pixel box size were then re-extracted using 288-pixel box size with an inter-box distance of 31 Å and particles comprising an entire helical crossover were selected for the following 3D classification. An initial 3D reference was de novo generated using selected particles after 2D classification by relion_helix_inimodel2d and the initial 3D model low-pass filtered to 60 Å was then applied further as a reference map to perform 3D classification. Local optimization of helical twist and rise was performed while β-strands perpendicular to the helical axis was clearly separated. The symmetry of psedo-2_1_ was applied and several rounds of 3D classification with *K* = 3 and *K* = 1 were used to gain segments belonging to the same conformation and optimize twist parameters. Optimized parameters and selected segments were applied for high-resolution gold-standard refinement. Post-processing with a soft-edge solvent mask in 30% central Z length was performed to sharpen the refined maps. The final overall resolution estimate was calculated to be 2.96 Å based on the 0.143 Fourier shell correlation cutoff. The model was built into the central region of the sharpened density map using E46K α-syn structure ((PDB entry code 6L4S) as an initial model in COOT^46^. A three-layer model was generated and refined by the real_space_refine program in PHENIX^47,48^.

### WT_51cs_ dataset

12,428 filaments were manually picked from 725 micrographs. WT_51cs_ segments were first extracted using 1200-pixel box size with an inter-box distance of 127 Å and used for several iterations of reference-free 2D classification. Filaments containing selected segments were then re-extracted using a 686-pixel box size with an inter-box distance of 73 Å and used for subsequent reference-free 2D classification. An initial 3D reference was de novo generated by relion_helix_inimodel2d using selected particles comprising an entire helical crossover and the initial 3D model low-pass filtered to 60 Å was then applied further as a reference map to perform de novo 3D classification. Local optimization of helical twist and rise was performed while β-strands perpendicular to the helical axis was clearly separated. Several rounds of 3D classification with *K* = 3 and *K* = 1 were then performed.

### WT_wts_ dataset

7903 filaments were manually picked from 1056 micrographs and first extracted with a 1024-pixel box size with an inter-box distance of 109 Å and used for several iterations of reference-free 2D classification. Filaments containing selected segments were then re-extracted using a 686-pixel box size with an inter-box distance of 73 Å. Similarly, with G51D and WT_51cs_ dataset, an initial 3D reference was de novo generated by relion_helix_inimodel2d using selected particles comprising an entire helical crossover, and the initial 3D model low-pass filtered to 60 Å was then applied further as a reference map to perform de novo 3D classification. Several rounds of 3D classification with *K* = 3 and *K* = 1 were then performed.

### Reporting summary

Further information on experimental design is available in the Nature Research Reporting Summary linked to this paper.

## Supplementary information

Supplementary information

Reporting summary

## Data Availability

Density maps of the G51D fibril are available through EMDB with entry code: EMD-30931. The structural model was deposited in the Protein Data Bank with entry code: 7E0F. Other structural models used in this study are available in the Protein Data Bank with entry codes: 6A6B (α-synuclein polymorph 1a fibril), 6L4S (E46K α-synuclein fibril). The source data underlying Figs. 1a, b, c, and 4b, c, d, Supplementary Figs. 1a, b, c, 3a, 4a, c, and 6a, b are provided as a Source Data file with this paper. Other data that support the findings of this study are available from the corresponding author upon reasonable request. Source data are provided with this paper.

## Source data

Source Data
