# Supplementary material for: The hereditary mutation G51D unlocks a distinct fibril strain transmissible to wild-type α-synuclein
Source: Nat Commun. 2021 Oct 29;12:6252. doi: 10.1038/s41467-021-26433-2 (PMC8556266; doi:10.1038/s41467-021-26433-2)
Supplement: Supplementary file 1 — Supplementary information [file 41467_2021_26433_MOESM1_ESM.pdf]

Supplementary Information

The hereditary mutation G51D unlocks a distinct fibril strain transmissible to wild-type α-synuclein

Yunpeng Sun[1, 2#], Houfang Long[1, 2#], Wencheng Xia[1, 2], Kun Wang[1, 2], Xia Zhang[3], Bo Sun[3], Qin Cao[4], Yaoyang Zhang[1, 2], Bin Dai[5], Dan Li[4], Cong Liu[1, 2*]

[1]Interdisciplinary Research Center on Biology and Chemistry, Shanghai Institute of Organic Chemistry, Chinese Academy of Sciences, Shanghai 201210, China;

[2]University of Chinese Academy of Sciences, Beijing 100049, China;

[3]School of Life Science and Technology, ShanghaiTech University, Shanghai, 201210, China;

[4]Bio-X Institutes, Key Laboratory for the Genetics of Developmental and Neuropsychiatric Disorders, Ministry of Education, Shanghai Jiao Tong University, Shanghai, 200030, China;

[5]Institute of Nano Biomedicine and Engineering, Department of Instrument Science and Engineering, School of Electronic Information and Electrical Engineering, Shanghai Jiao Tong University, Shanghai, 200210, China

#These authors contributed equally to this work.

* To whom correspondence should be addressed. E-mail: liulab@sioc.ac.cn

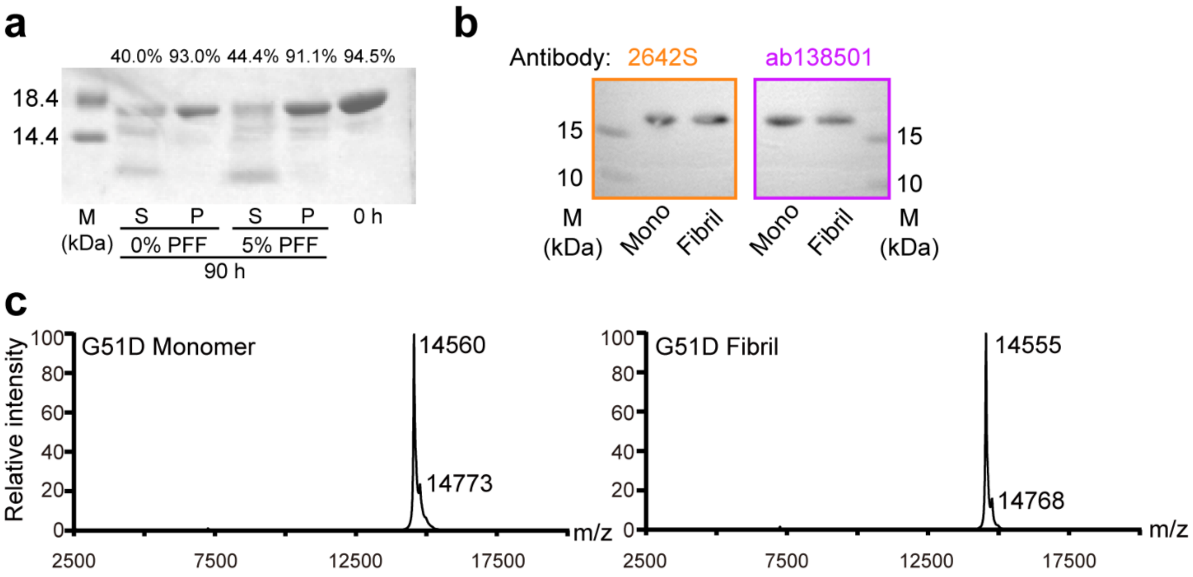

Supplementary Figure 1. Purity of full-length G51D α-syn in the fibrils.

a, SDS-PAGE gel of the samples from the ThT kinetic assay. Supernatant (S) and pellet (P) of the samples at the end point (90 h) and at the start point (0 h) were loaded on the gel. M, protein marker. Intensities of the protein bands were analyzed by Image Lab 3.0 (Bio-Rad). Percentage of the full-length G51D protein band in each lane is marked. The result is reproducible in 3 independent experiments. **b**, Western blots of G51D fibrils with α-syn antibodies 2642S (polyclonal antibody) and ab138501 (monoclonal antibody recognizing residues 118-123). Monomer is loaded as a control. The result is reproducible in 3 independent experiments. **c**, MALDI-TOF mass spectra of G51D monomer and fibril in the range (m/z) 2500–20000.

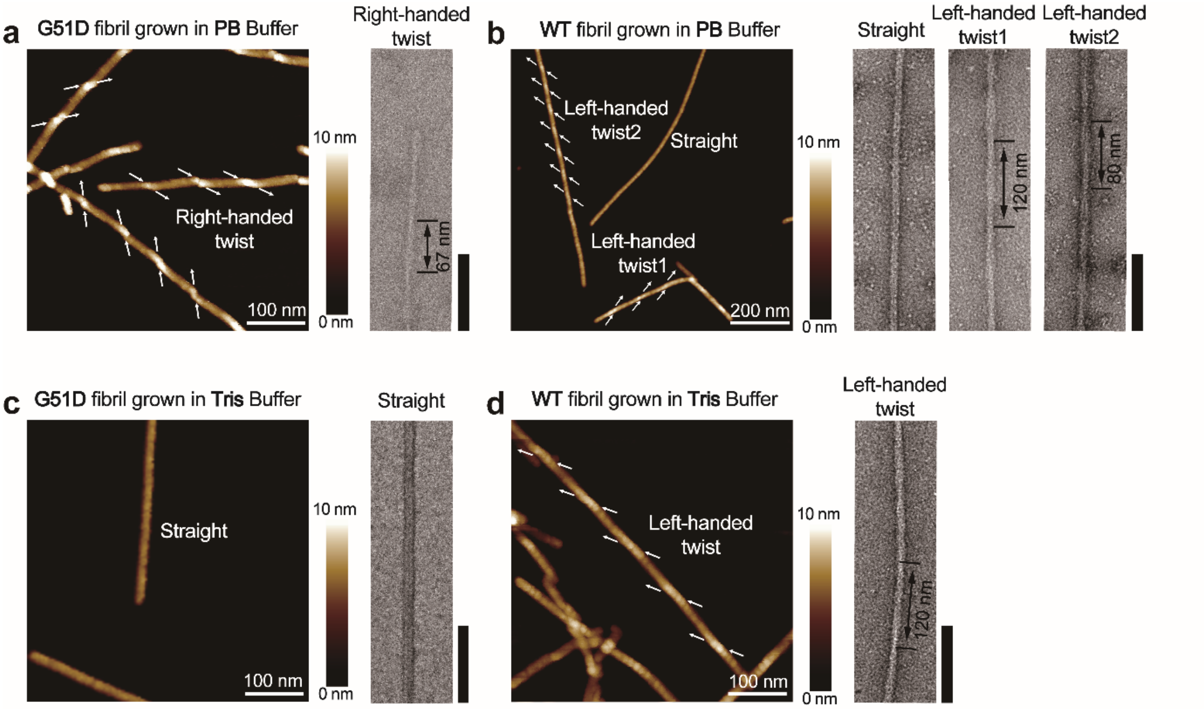

e

Fibril	Tris Buffer		PB Buffer	
	Chirality	Half pitch (nm)	Chirality	Half pitch (nm)
WT	Left-handed	120.6 ± 7.7	Straight (93.8%)	——
			Twist1: Left-handed (3.6%)	118.9 ± 5.8
			Twist2: Left-handed (2.6%)	78.0 ± 4.6
G51D	Straight	——	Right-handed	67.0 ± 9.6

Supplementary Figure 2. Characterization of G51D fibrils formed in Tris buffer and WT fibrils formed in PB buffer. a, AFM (left) and negative-staining TEM (right) images of the G51D fibrils formed in PB Buffer. **b,** AFM and TEM images of the WT fibrils formed in PB Buffer. **c,** AFM and TEM images of the G51D fibrils formed in Tris buffer. **d,** AFM and TEM images of the WT fibrils formed in Tris Buffer. Scale bars of TEM images in (**a-d**) are 100 nm. Arrows in AFM images at both sides of a fibril indicate starting points of the protrusion to clarify the handedness. **e,** List of the helical parameters of WT fibrils formed in Tris buffer (n= 90 fibrils), WT fibrils in PB buffer (n= 305 fibrils), G51D fibrils in Tris buffer (n= 80 fibrils), and G51D fibrils in PB buffer (n= 83 fibrils), respectively. Half pitches were measured based on the TEM images. Values are means ± SD. Percentages of each type of WT fibril polymorphs formed in PB Buffer are provided in parentheses (n = 305 fibrils).

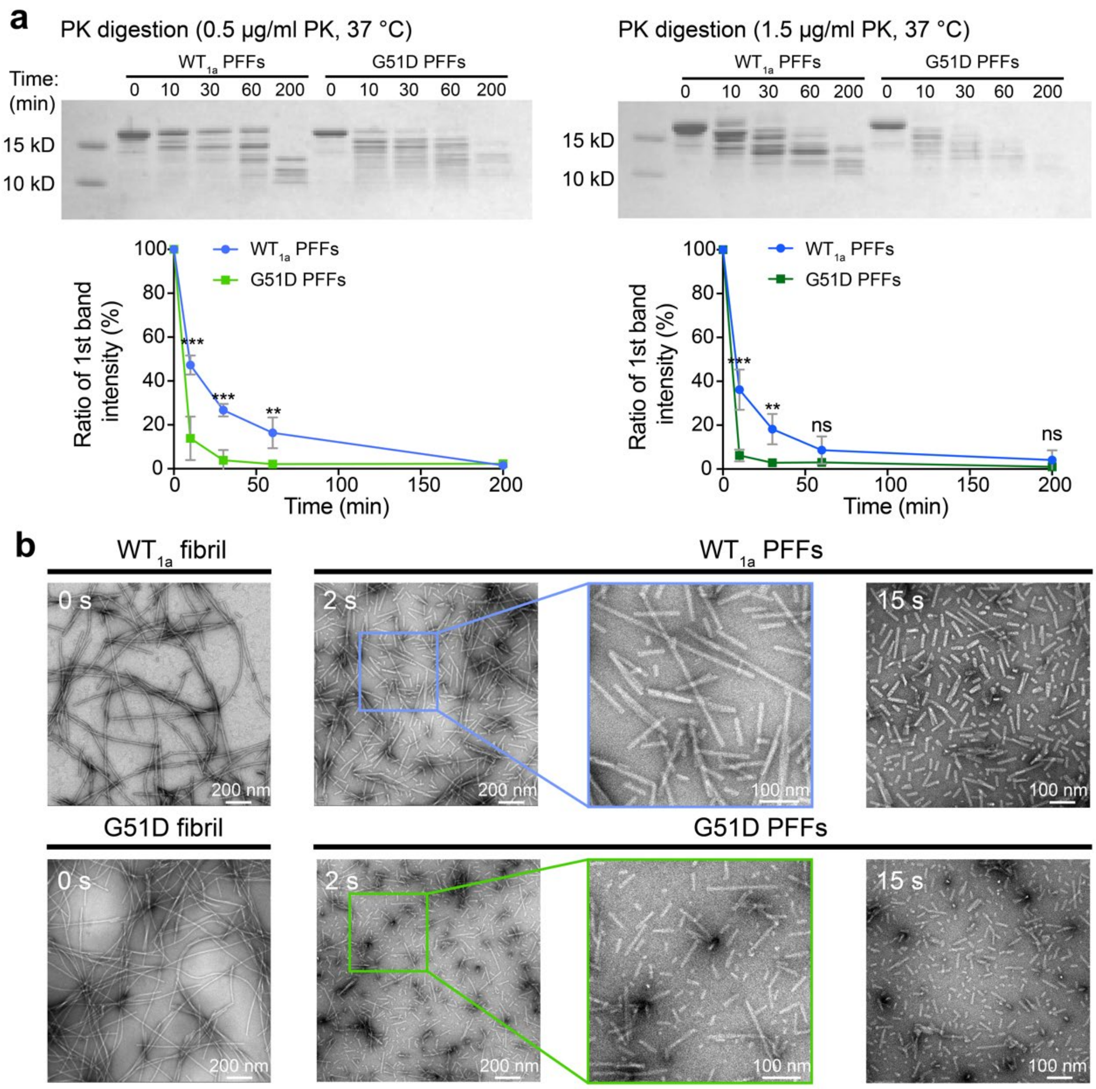

Supplementary Figure 3. Comparison of the stability of the WT_{1a} and G51D α-syn fibrils. **a,** PK digestion of WT and G51D fibrils. The fibrils were incubated with 0.5 μg/ml (left) and 1.5 μg/ml (right) of PK at 37 °C for 10 min, 30 min, 60 min and 200 min. Intensities of the first protein band (representing full-length protein) in each lane on SDS-PAGE are analyzed (bottom). Data shown are mean ± s.d., n = 3 independent samples. The level of significance was set as $**p$ <0.01(0.5 μg/ml PK, 60min, WT_{1a} vs. G51D PFFs: 0.0074; 1.5 μg/ml PK, 30min WT_{1a} vs. G51D PFFs: 0.0051); $***p$ <0.001(0.5 μg/ml PK, 10min WT_{1a} vs. G51D PFFs: 0.0008; 0.5 μg/ml PK, 30min, WT1a vs. G51D PFFs: 0.0002; 1.5 μg/ml PK, 10min WT_{1a} vs. G51D PFFs: 0.0007); ns, not significant. P-values are based on two-sided Student's t-test. **b,** NS-TEM images of 5 μM WT_{1a} (top) and G51D (bottom) α-syn PFFs after sonication. The images represent reproducible results in three independent experiments.

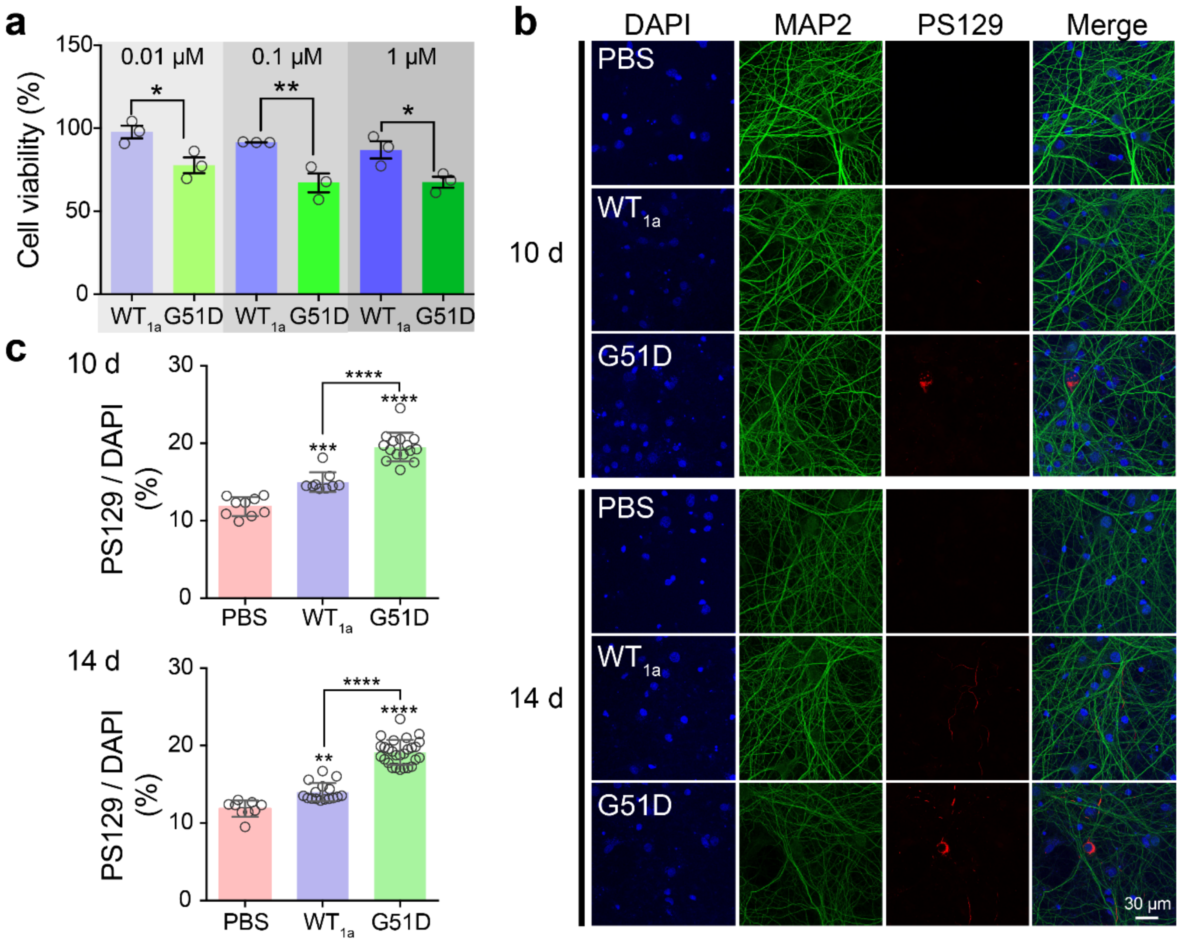

Supplementary Figure 4. Comparison of the toxicity and propagation of the WT_{1a} and G51D α-syn fibrils. **a,** Cellular toxicity of G51D and WT_{1a} PFFs to SH-SY5Y cells is tested by using the CCK-8 kit. Data shown are mean ± s.d., n = 3 independent samples. The level of significance was set as * p<0.05(0.01 μM WT_{1a} vs. 0.01 μM G51D: 0.0173; 1 μM WT_{1a} vs. 1 μM G51D: 0.0208); ** p<0.01(0.1 μM WT_{1a} vs. 0.1 μM G51D: 0.0046) for one-way ANOVA followed by Tukey honestly significant difference (HSD) post hoc test. The plot was generated by GraphPad Prism 6. **b,** Confocal images of rat primary neurons treated with PBS (blank control), 100 nM WT1a PFFs, and G51D PFFs for 10 d and 14 d, respectively. The fixed neurons were immunostained for DAPI (blue), pS129 α-syn (red) and microtubule-associated protein 2 (MAP2) (green). Scale bar: 30 μm. The intensity of confocal images was analyzed by Image J 2.0.0. **c,** Quantitative analysis of PS129 α-syn aggregation in (**b**). The intensity of PS129 was normalized to the signal of DAPI for each sample. Statistical significance was measured by one-way ANOVA. Data shown are mean ± s.d., n = 3 independent samples. Every dot represents an image. The level of significance was set as ** p<0.01(14d WT_{1a} vs. PBS: 0.0020); *** p<0.001(10d WT_{1a} vs. PBS: 0.0005), **** p<0.0001(10d G51D vs. PBS: <0.0001; 10d G51D vs. WT_{1a}: <0.0001; 14d G51D vs. PBS: <0.0001; 14d G51D vs. WT_{1a}: <0.0001) for one-way ANOVA followed by Tukey HSD post hoc test.

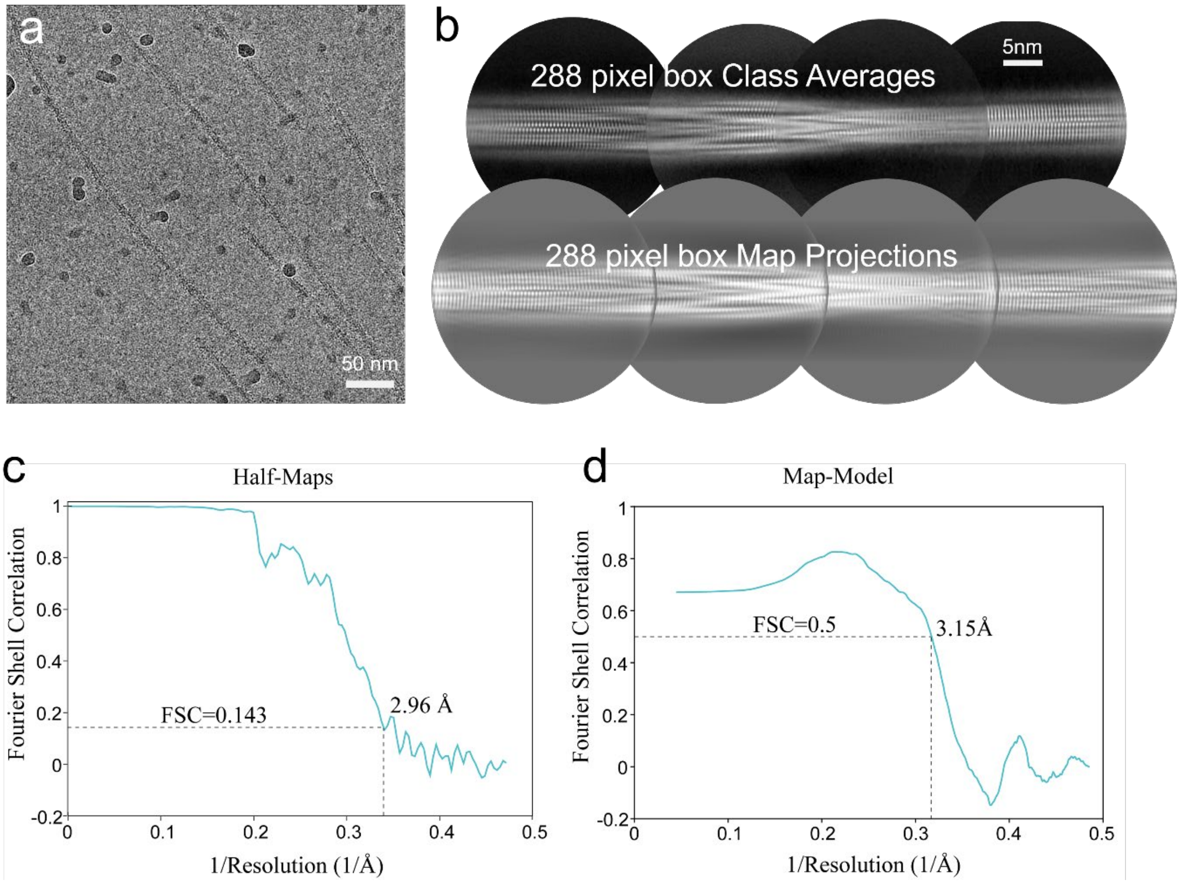

Supplementary Figure 5. Cryo-EM data collection, reconstruction, and refinement of G51D α-syn fibrils. a, Cryo-EM micrograph of G51D α-syn fibrils. Scale bar, 50 nm. Fibrils formed by G51D in >3 independent experiments provide reproducible images. **b,** 2D class averages used to calculate crossover distance and map projections match 2D class averages. Box size: 288 pixels. Scale bar, 5 nm. **c,** Fourier shell correlation curve between half maps. **d,** Fourier shell correlation curve between cryo-EM reconstruction map and the refined model.

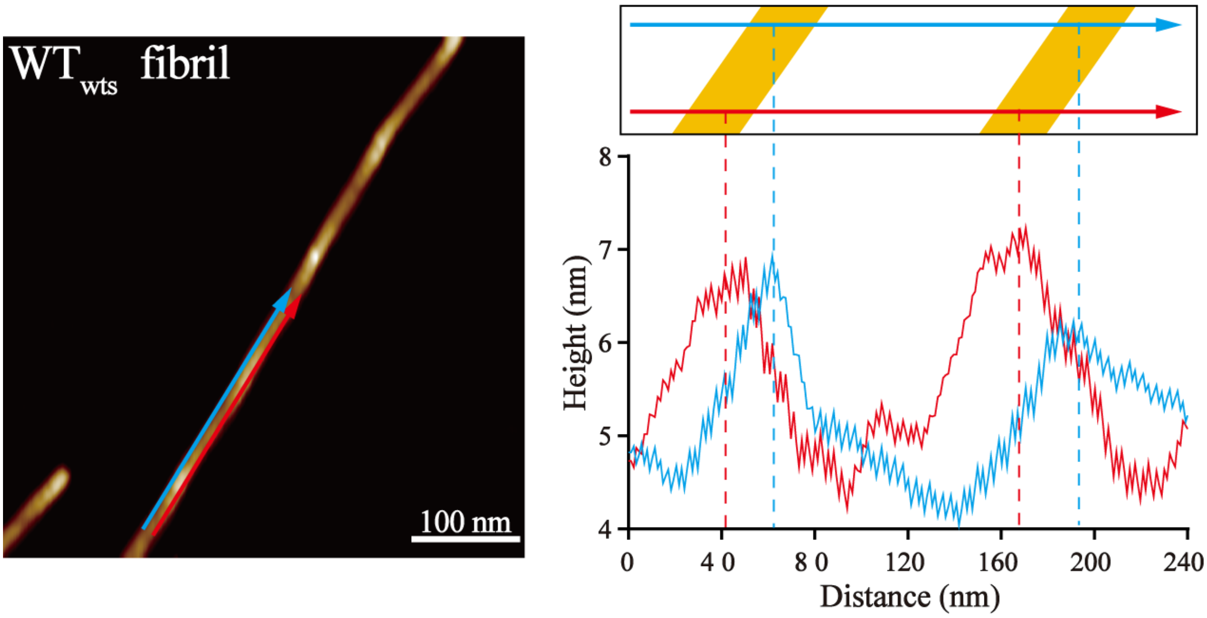

Supplementary Figure 6. Handedness analysis of the of the WT_{wts} fibrils.

Overlay of two height profiles of both sides along a WT_{wts} fibril. Blue and red lines represent height profiles (from the bottom to the top) at the left side and right side, respectively. Yellow blocks represent the protrusion along a fibril.

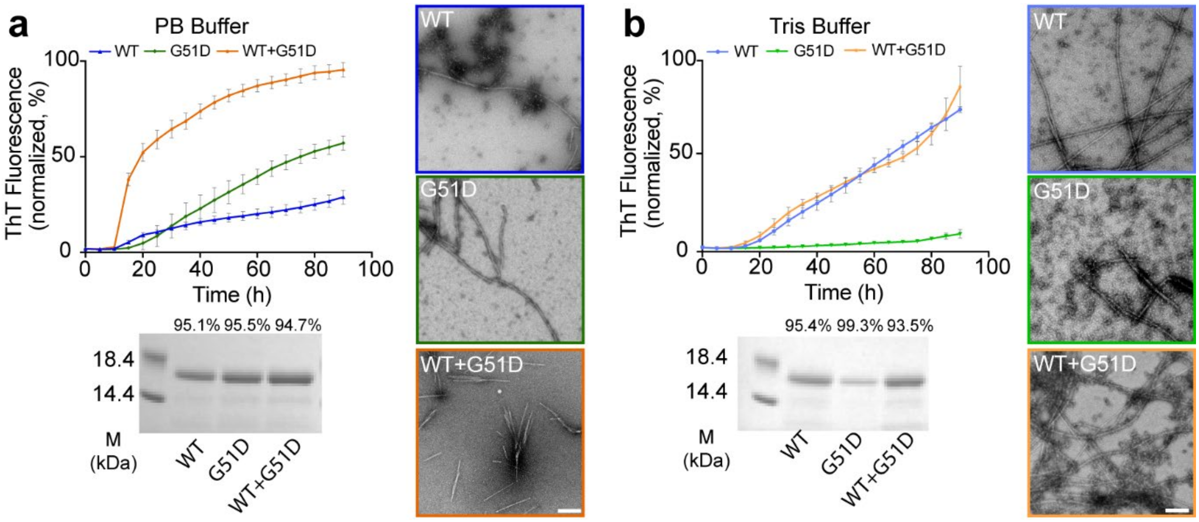

Supplementary Figure 7. Co-aggregation of WT and G51D α-syn.

a, Co-aggregation of WT and G51D α-syn in PB buffer. **b**, Co-aggregation of WT and G51D α-syn in Tris buffer. In each panel, ThT kinetic assay is shown on top left. Data are shown as mean ± s.d., n=3 independent samples. SDS-PAGE of ThT samples at the end time point is shown at bottom left. M, protein marker. Intensities of the protein bands were analyzed by Image Lab 3.0 (Bio-Rad). Percentage of the full-length protein bands are marked. Fibrils formed at the end time point in the ThT assay were characterized by TEM (right). Scale bar: 200 nm. The result is reproducible in 3 independent experiments.

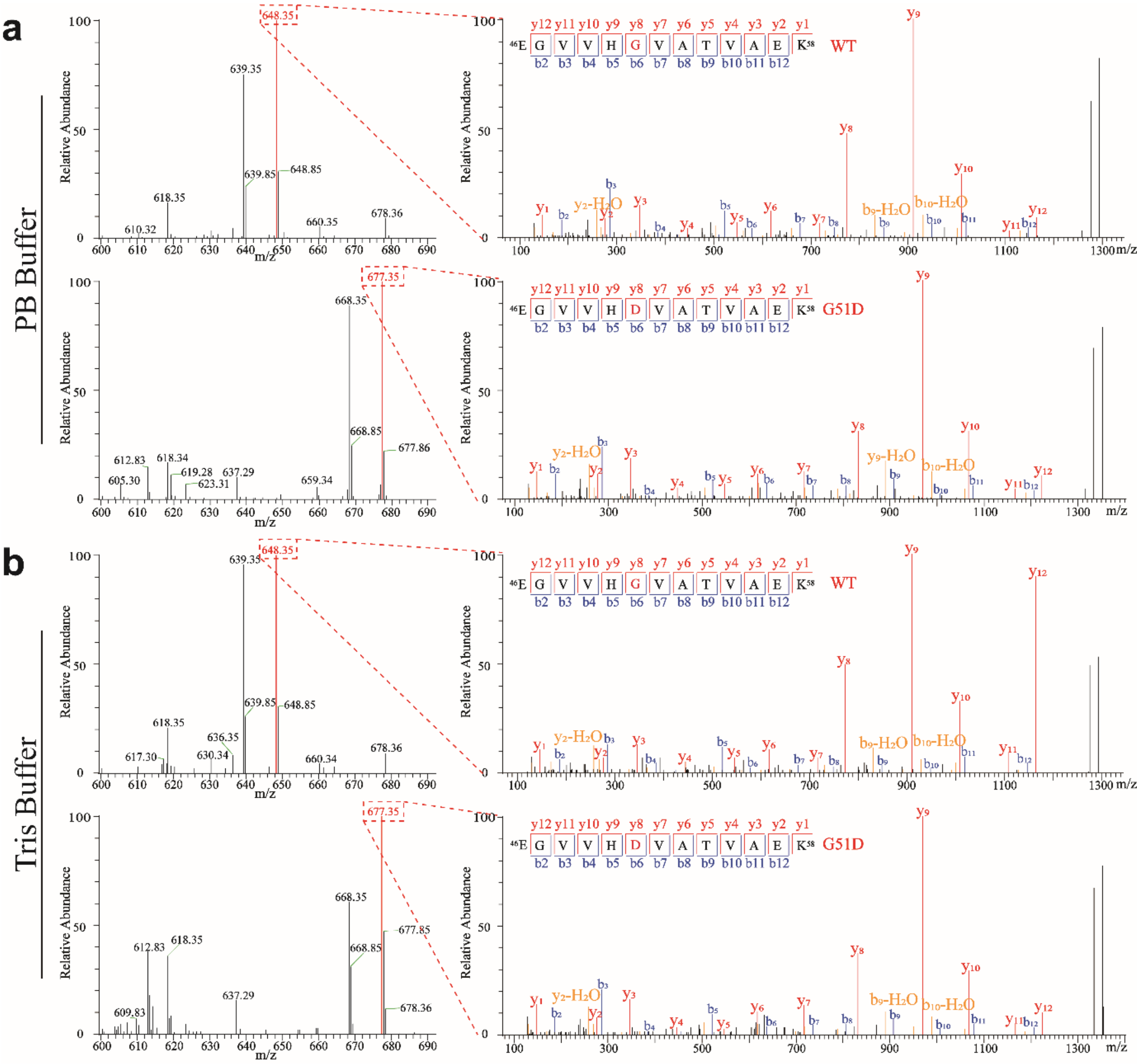

Supplementary Figure 8. MS validation of the WT+G51D fibrils formed in Tris Buffer and PB buffer. LC-MS/MS analysis of WT+G51D monomer and dissolved fibrils formed in Tris Buffer (**a**) and PB buffer (**b**). Peptide ^{46}EGVVHGVATVAEK^{60} or ^{46}EGVVHDVATVAEK^{60} is identified in the chromatograms of peptide mixture (left panel), as peptide ^{46}EGVVHGVATVAEK^{60} from trypsin digestion represents WT α-syn and peptide ^{46}EGVVHDVATVAEK^{60} from trypsin digestion represents G51D α-syn. MS/MS spectra (right panel) containing fragments with b - or y -type ions confirmed sequence of these two peptides, respectively.

Supplementary Table 1 List of the primers used in this work.

Primers for G51D point mutation	
Sequence (5’-3’)-F	TGCATGATGTGGCAACAGTGGCTGAG
Sequence (5’-3’)-R	CCACATCATGCACCACTCCCTCC
